# An improved contrastive learning network for semi-supervised multi-structure segmentation in echocardiography

**DOI:** 10.3389/fcvm.2023.1266260

**Published:** 2023-09-22

**Authors:** Ziyu Guo, Yuting Zhang, Zishan Qiu, Suyu Dong, Shan He, Huan Gao, Jinao Zhang, Yingtao Chen, Bingtao He, Zhe Kong, Zhaowen Qiu, Yan Li, Caijuan Li

**Affiliations:** ^1^ College of Computer and Control Engineering, Northeast Forestry University, Harbin, China; ^2^ School of Computer Science, University of Birmingham, Birmingham, United Kingdom; ^3^ College of Art and Science, New York University Shanghai, Shanghai, China; ^4^ Department of Medical Ultrasonics, Hongqi Hospital of Mudanjiang Medical University, Mudanjiang, China

**Keywords:** echocardiography, deep learning, semi-supervised learning, images semantic segmentation, contrastive learning

## Abstract

Cardiac diseases have high mortality rates and are a significant threat to human health. Echocardiography is a commonly used imaging technique to diagnose cardiac diseases because of its portability, non-invasiveness and low cost. Precise segmentation of basic cardiac structures is crucial for cardiologists to efficiently diagnose cardiac diseases, but this task is challenging due to several reasons, such as: (1) low image contrast, (2) incomplete structures of cardiac, and (3) unclear border between the ventricle and the atrium in some echocardiographic images. In this paper, we applied contrastive learning strategy and proposed a semi-supervised method for echocardiographic images segmentation. This proposed method solved the above challenges effectively and made use of unlabeled data to achieve a great performance, which could help doctors improve the accuracy of CVD diagnosis and screening. We evaluated this method on a public dataset (CAMUS), achieving mean Dice Similarity Coefficient (DSC) of 0.898, 0.911, 0.916 with 1/4, 1/2 and full labeled data on two-chamber (2CH) echocardiography images, and of 0.903, 0.921, 0.928 with 1/4, 1/2 and full labeled data on four-chamber (4CH) echocardiography images. Compared with other existing methods, the proposed method had fewer parameters and better performance. The code and models are available at https://github.com/gpgzy/CL-Cardiac-segmentation.

## 1. Introduction

Cardiovascular diseases (CVDs) are increasing threats to global health and have become the leading cause of death in industrialized countries (1). The American Society of Echocardiography (ASE) and the European Association of Cardiovascular Imaging (EACVI) have emphasized the importance of cardiac chamber quantification by echocardiography in the diagnosis and treatment of cardiovascular diseases (2). Echocardiography is one of the most widely utilized diagnostic tests in cardiology, offering clear visualizations of left ventricular size during end systole and end diastole, along with the thickness of the myocardium (3). In echocardiography, a heart afflicted by disease might display enlarged atrial and ventricular volumes or an augmented thickness of the myocardium (4). However, these chamber quantifications, such as chamber sizes, volumes, and etc., are usually based on precise segmentation of certain critical structures (such as ventricle, atrium and myocardium), and then measuring key metrics that indicate the heart’s functionality (2, 5). Practically, this process requires cardiologists to manually describe the anatomy and takes measurements of relevant biological parameters, which can be tedious, time-consuming, and subjective (5). Therefore, there exists a genuine requirement in clinical settings for an efficient and precise automated echocardiographic segmentation technique that can enhance the efficacy and reduce the burden of the physician in clinical imaging screening, track disease progression and make informed decisions about treatment and intervention.

There are two main categories of automated segmentation techniques for cardiac structures: traditional techniques and neural network-based methods. Traditional methods include contour models (6), level sets (7), and atlas-based methods (8). Barbosa et al. (6) put forward a B-spline active contour formulation that employs explicit functions for real-time segmentation of 3D echocardiography and liver computer tomography. This method overcomes the limitations of the initial Active Geometric Functions (AGF) framework introduced by Real-time segmentation by Active Geometric Functions while preserving computational speed. Yang et al. (7) proposed a two-layer level set method along with a circular shape constraint to segment the left ventricle (LV) from short-axis cardiac magnetic resonance images (CMRI) without relying on any pre-trained models. This technique can be applied to other level set methods and effectively addresses common issues in LV segmentation, such as intensity overlap between Trabeculations and Papillary Muscles (TPM) and the myocardium, and the existence of outflow track in basal slices. Zhuang et al. (8) developed a fully automated framework for whole-heart segmentation that relies on the locally affine registration method (LARM) and free-form deformations with adaptive control point status (ACPS FFDs) for automatic segmentation of CMRI. However, these methods are unable to surmount the challenges of low contrast and noise that are inherent in echocardiography. As a result, they are unable to produce accurate segmentation results based on echocardiography.

Neural network-based methods have demonstrated enhanced segmentation accuracy in echocardiography as well, and they can be classified further as supervised methods and semi-supervised methods. Cui et al. (9) proposed a multitask model with Task Relation Spatial Co-Attention for joint segmentation and quantification on 2D echocardiography. This method integrated the Boundary-aware Structure Consistency (BSC) and Joint Indices Constraint (JIC) into the multitask learning optimization objective to guide the learning of segmentation and quantification paths. It was validated on the CAMUS dataset and demonstrated outstanding performance, achieving an overall mean Dice score of 0.912 and 0.923, as well as average precision scores of 0.931 and 0.941 for the two-chamber views (A2C) and apical four-chamber views (A4C). Cui et al. (10) utilized a training strategy named multi-constrained aggregate learning (MCAL) for the segmentation of myocardium in 2D echocardiography. This method leveraged anatomical knowledge learned through ground-truth labels to infer segmented parts and discriminate boundary pixels. It was validated on CAMUS dataset and had performance with segmentation Dice of 0.853±0.057 and 0.859±0.560 for the apical A4C and A2C views, respectively. Hamila et al. (11) proposed a novel convolution neural network (CNN) that combines denoising and feature extraction techniques for automatic LV segmentation of echocardiography. 2D echocardiographic images from 70 patients were used to train this network, and it was then tested on 12 patients, achieving a segmentation Dice of 0.937. While these supervised methods can achieve excellent performance, they all require a sufficient number of pixel-wise annotations to train the model, which can be a time-consuming and tedious process.

Semi-supervised methods have shown effectiveness in reducing the need for a large number of annotated samples in echocardiography segmentation. Wu et al. (5) integrated a novel adaptive spatiotemporal semantic calibration module into the mean teacher semi-supervised architecture to determine spatiotemporal correspondences based on feature maps for echocardiography segmentation. The proposed method was evaluated using the EchoNet-Dynamic and CAMUS datasets, resulting in average Dice coefficients of 0.929 and 0.938, respectively, for the segmentation of the left ventricular endocardium. Additionally, based on these two datasets, El Rai et al. (12) presented a new semi-supervised approach called GraphECV for the segmentation of the LV in echocardiography by using graph signal processing, respectively resulting in Dice coefficients of 0.936 and 0.940 with 1/2 labeled data for the left ventricular segmentation. Wei et al. (13) used a co-learning mechanism to explore the mutual benefits of cardiac segmentation, therefore alleviating the noisy appearance. It was validated on the training set of CAMUS dataset using 10-fold cross-validation, achieving a Dice of 0.923, 0.948 and 0.895 for the segmentation of LV, myocardium and left atrium (LA). Chen et al. (14) proposed a framework for cross-domain echocardiography segmentation that incorporated multi-space adaptation-segmentation-joint based on a generative adversarial architecture with a generator and multi-space discriminators. The CAMUS dataset was used to evaluate this method, and the experiments show that this method attained the mean Dice coefficients of 0.890 for the segmentation of LV endocardium and LV epicardium. However, only a few semi-supervised methods have been used for the segmentation of the LV, LA, and myocardium, and although some methods attempt to segment all three regions simultaneously, the accuracy of multi-structure segmentation remains to be improved, especially for the LV and LA segmentation.

To help improve the accuracy of diagnosing and screening for cardiovascular diseases while also easing the workload associated with evaluating LV images. In this paper, we proposed a novel semi-supervised method for multi-structure segmentation of echocardiography, and the main contributions could be summarized as follows:

(1) We first applied contrastive learning in the multi-structure segmentation of echocardiography, and could accurately segment LV, LA and myocardium without requiring a large number of annotated samples, which explored the feasibility of contrastive learning in echocardiography multi-structure segmentation.
(2) We made two improvements to the existing model, building upon the work by Lai et al. (15): replacing DeeplabV3+ (16) with u-net (17) and modifying the structure of the projector. These changes aimed to tackle challenges in echocardiography, such as low contrast, unclear boundaries, and incomplete cardiac structures.
(3) Our method was evaluated on the CAMUS dataset, and it demonstrated excellent performance in both two-chamber (2CH) and four-chamber (4CH) echocardiographic images.

## 2. Method

### 2.1. Overview

**Figure 1:**
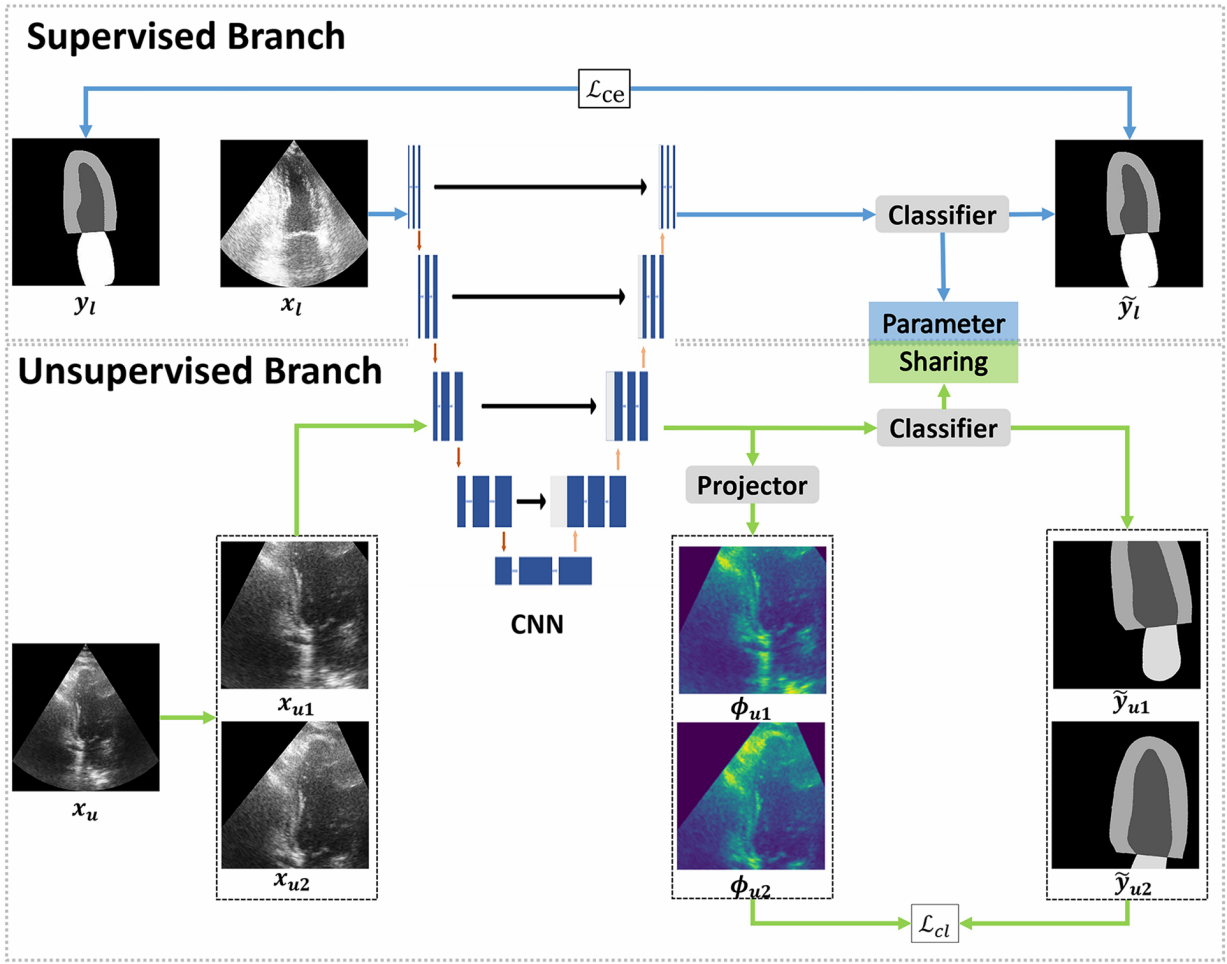
Overview of the proposed framework. The proposed framework can be divided into two branches: supervised and unsupervised. The supervised branch was trained with a few epochs firstly, followed by training the unsupervised branch with a lot of epochs. The supervised branch consists of a convolutional neural network (CNN) to extract the feature map of labeled images and a classifier to make predictions. In the unsupervised branch, the same CNN and classifier from the supervised branch were used, the classifiers in two branches share parameters with each other. In CNN, each blue box represents a multi-channel feature map and each white box represents a copied feature map. The downward red arrows indicate the downsampling stages and the upward red arrows indicate the upsampling stages. The blue arrow to the right represents a convolution of 1×1 and the black arrow to the right indicates a skip connection. The dilated convolutions were implemented in the downsampling stages. Furthermore, a projector was introduced to modify the feature dimensions. In the supervised branch, the loss function employed a standard cross-entropy loss ($L_{ce}$), which measures the discrepancy between the predictions and the ground truth. On the other hand, the unsupervised branch employed a contrastive learning loss ($L_{cl}$) to quantify the difference between pseudo labels and feature maps.

The cardiac structure segmentation network in this study was built upon the contrastive learning, taking inspiration from the work of Lai et al. (15) (Figure 1). The proposed network consists of two branches: a supervised branch and an unsupervised branch. The supervised branch was first trained with labeled data to acquire basic features within the echocardiographic images. Next, these parameters were shared with the unsupervised branch, which was then continually optimized with unlabeled data. More details about the supervised branch, the unsupervised branch and loss functions were introduced specifically in Sections 2.2, 2.3 and 2.4.

### 2.2. Supervised branch

The supervised branch of our network, like other supervised networks, was composed of a convolutional neural network (CNN) and a classifier. The CNN was employed to extract features from images, whereas the classifier was responsible for mapping these features to predictions. More specifically, we employed the u-net as the backbone in supervised branch to convert training images into feature vectors, which was the CNN referred to in Figure 1. U-net (17) is a type of neural network that follows an encoder-decoder structure, where the encoder is able to capture context information and the decoder can perform precise localization. The encoder and decoder are connected through a skip connection. While the skip connection in u-net can help prevent shallow features from being lost, the use of multiple pooling layers in the contracting path can result in information loss in the images. Dilated convolutions can be used to solve this issue by increasing the field of perception without adding more parameters, minimizing information loss during downsampling. These convolutions inflate the kernel with holes between the kernel elements, and the dilation rate parameter indicates the amount the kernel is widened (18). To enhance the performance of the proposed network, we incorporated dilated convolutions into the downsampling process of u-net, and used this modified u-net as the CNN. The dilated convolutions were implemented in the second, third and fourth downsampling stages of u-net, with dilation rates of 1, 2 and 4 respectively. To evaluate the effectiveness of the dilated convolutions, we conducted an ablation experiment which was described in Section 3.6.

### 2.3. Unsupervised branch

The proposed unsupervised branch was based on the contrastive learning strategy, which is a type of framework for learning discriminative representations. The main focus of contrastive learning was to compare pairs of sample examples that are considered to be either similar (positive samples) or dissimilar (negative samples) in terms of their semantic content.

In our work, each image was randomly cropped twice and then done some different augmentations to create two different transformation views, which were considered as positive samples. In this process, we made sure the two positive samples have an overlap region. The negative samples were images from the training set, but without including the given image. As shown in Figure 1, we transformed the $x_{u}$ image to create $x_{u1}$ and $x_{u2}$, which serve as positive samples, and randomly selected images from our training set (excluding $x_{u}$) as negative samples. The training process aimed to bring the positive samples ($x_{u1}$ and $x_{u2}$) closer together and separated the negative samples that belong to other classes. To maintain consistency in the representation of the overlap region, we employed the loss function $L_{cl}$. Finally, the unsupervised branch was able to learn the deep features of ventricle, myocardium and atrium and discriminate them well without labeled images.

**Figure 2:**
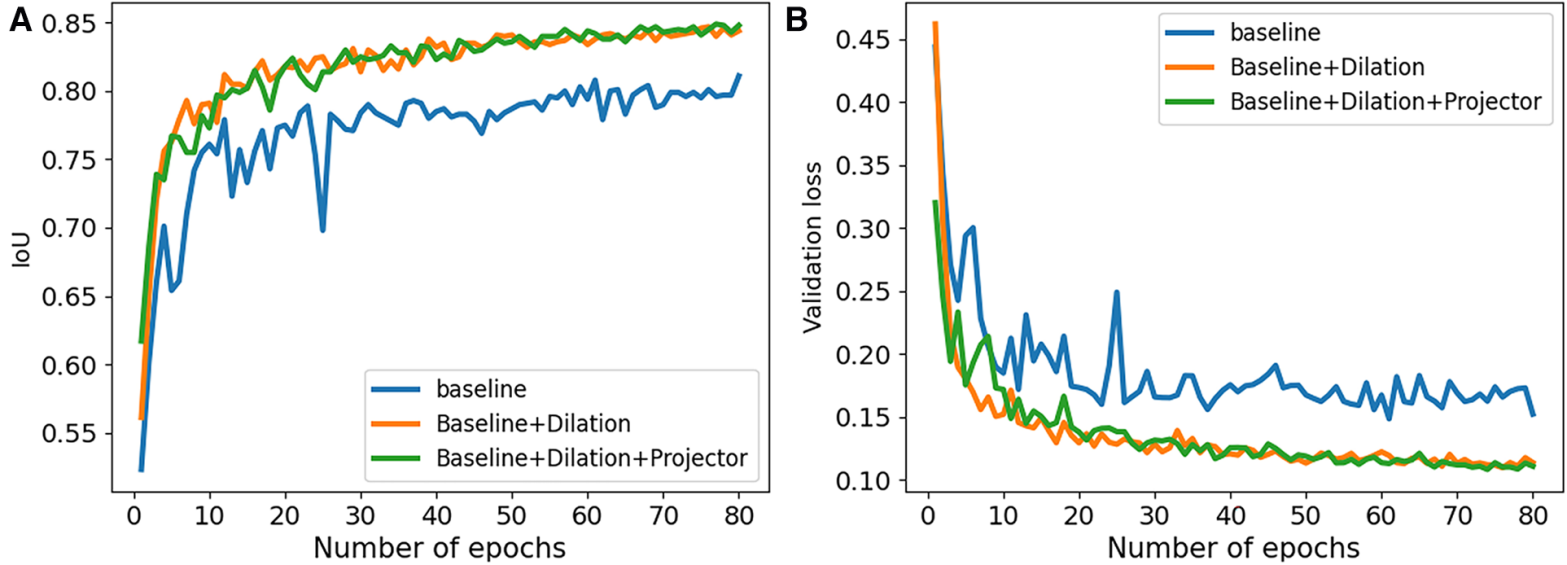
Performance comparison of our ablation studies on 2CH images from CAMUS dataset. (**A**) The IoU values were examined to assess the performance of our ablation studies. (**B**) The validation loss values were analyzed to evaluate the performance of our ablation studies.

As shown in Figure 2, the unsupervised branch consisted of a CNN, a classifier and a projector. Among them, the CNN is the same one shared with the supervised branch. The classifier in unsupervised branch had the same architecture as that in the supervised branch, and they shared the same parameters. The projector was comprised of two linear layers, with the first layer followed by batch normalization and rectified linear units (ReLU). The purpose of the projector was to change the feature dimension and prevent the loss of useful information for segmentation. An ablation experiment was performed to evaluate the significance of this projector, and more details have been shown in Section 3.6. The loss function was a pixel-wise contrastive loss, and it will be elaborated upon, as shown in Section 2.4.

### 2.4. Loss functions

#### 2.4.1. Supervised loss

Cross entropy loss was used in supervised networks to measure the dissimilarity between the predicted probability distribution of class labels and the actual distribution of class labels (19). Compared with other loss functions, this loss function is differentiable and easy to optimize using gradient based methods. Therefore, in the supervised branch, we used cross entropy loss as the loss function, which is frequently employed for image semantic segmentation tasks.

The cross-entropy loss $L_{ce}$ can be written as follows:

(1) $$L_{ce}=-\frac{1}{N}\sum_{i}\sum_{c=1}^{M}y_{ic}log(p_{ic})$$

where M and N are the number of classes and samples; $y_{ic}$ depends on the truth value of i, if it is equals to c, $y_{ic}$ is 1, else $y_{ic}$ is 0. $p_{ic}$ is the probability of sample i belongs to class c.

#### 2.4.2. Unsupervised loss

In the unsupervised branch, followed the previous work Lai et al. (15), the proposed loss function $L_{cl}$ is:

(2) $$L_{cl}=L_{ce}+\lambda L_{dc}^{ns,pf}$$

where λ is used to control the contribution of $L_{dc}^{ns,pf}$, and the range of λ is 0-1. In our experiment, we set λ with 0.1. $L_{ce}$ is a standard cross entropy loss, which is the same as the loss function in supervised branch.

$L_{dc}^{ns,pf}$ was a Directional Contrastive loss (DC Loss), which was used to minimize the distance between positive feature pairs (features with the same class) and maximize the distance between negative feature pairs (features with different classes). Specifically, as shown in Figure 1, we regarded two features of $\phi_{u1}$ and $\phi_{u2}$ as a positive pair, because both of them corresponded to the same pixels in $x_{u}$ but with different transformations. In addition, any two images in the training dataset were regarded as a negative pair.

The DC Loss ($L_{dc}^{ns,pf}$) can be written as follows:

(3) $$L_{dc}^{ns,pf}=\frac{1}{B}\sum_{b=1}^{B}(l_{dc}^{b,ns,pf}(\phi_{u1},\phi_{u2})+l_{dc}^{b,ns,pf}(\phi_{u2},\phi_{u1}))$$

where B represents the batch size of training.

The $l_{dc}^{b,ns,pf}$ can be written as follows:

(4) $$l_{dc}^{b,ns,pf}(\phi_{u1},\phi_{u2})=-\frac{1}{N}\sum_{h,w}M_{d,pf}^{h,w}\cdot log\frac{r(\phi_{u1}^{h,w},\phi_{u2}^{h,w})}{r(\phi_{u1}^{h,w},\phi_{u2}^{h,w})+\sum_{\phi_{n}\in F_{u}}M_{n,1}^{h,w}\cdot r(\phi_{u1}^{h,w},\phi_{n})}$$

where $M_{d,pf}^{h,w}$ is the binary mask and can filter those uncertain positive samples. r denotes the exponential function of the cosine similarity s between two features with a temperature τ. $r(\phi_{1},\phi_{2})=\exp(s(\phi_{1},\phi_{2})/\tau )$; h and w denote the height and width of 2-D images; N denotes the number of spatial locations of $x_{u}$; $\phi_{n}\in R^{c}$ represents the negative counterpart of the feature $\phi_{u1}^{h,w}$, and $F_{u}$ represents the set of negative samples.

The binary mask $M_{d,pf}^{h,w}$ can be written as follows:

(5) $$M_{d,pf}^{h,w}=M_{d}^{h,w}\cdot 1\{maxC(f_{u2}^{h,w})>\gamma \}$$

where γ is a threshold. If the confidence of a positive sample is lower than γ, this positive pair will not contribute to the final loss.

The directional mask $M_{d}^{h,w}$ can be written as:

(6) $$M_{d}^{h,w}=1\{maxC(f_{u1}^{h,w})<maxC(f_{u2}^{h,w})\}$$

where C is the classifier. $f_{u1}^{h,w}$ and $f_{u2}^{h,w}$ are features of $x_{u1}$ and $x_{u2}$ extracted by CNN.

## 3. Experiment

### 3.1. Dataset

In this paper, the proposed method was validated on CAMUS dataset (20), which consisted of clinical exams from 500 patients. For each patient, it included two-dimension (2D) apical 2CH and 4CH view echocardiogram sequences, along with annotations for LV, LA, and LV endocardium at end diastole (ED) and end systole (ES) frames. Thereinto, 450 patients were used as the training dataset to train the proposed model, and 50 patients were used as the testing dataset to evaluate the performance of the trained model. For the training dataset, it contained 366 patients with good or medium image quality, and 84 patients with poor image quality. The testing dataset contained 40 patients with good or medium image quality, and 10 patients with poor image quality. Since the images in the dataset had different sizes, we resized all of them to a uniform resolution of 512×512 and normalized them to the range of [−1,1] before training.

### 3.2. Implementation details

The proposed network was implemented based on pytorch1.13 and trained on a single NVIDIA Tesla A40 GPU with 48GB memory. In order to reduce the GPU memory usage and improve the efficiency of training, we used automatic mixed precision (AMP) in training.

In our experiments, we initialized the network parameters randomly and opted for the SGD optimizer with a weight decay of 0.0001 and an initial learning rate of 0.01. To update the learning rate, we employed the poly decay policy, which can be expressed as follows:

(7) $$l(iter)=lr\cdot {\left(1-\frac{iter}{\mathrm{total}}\right)}^{\;\mathrm{power}}$$

where power=0.9; iter is the number of epochs we are currently training; total is the sum of the epochs used for training. We trained the supervised branch in the first 5 epochs before training the unsupervised branch. In the end, we completed training for a total of 80 epochs.

### 3.3. Data augmentation

In order to avoid the overfitting and improve the robustness of the proposed network, several data augmentations were applied before training, including Gaussian blur, color jitter, gray scale, horizontal flipping. In unsupervised branch, we applied random crop and random rotation. Specifically, we randomly cropped images to a size of 320×320 and rotated them with an arbitrarily degree within the range of $\left[-15^{\circ },15^{\circ }\right]$.

### 3.4. Training process

The training process of the supervised branch can be described as follows. Firstly, the labeled image $x_{l}$ was processed by the CNN (ε) to obtain its corresponding feature map $f_{l}=\varepsilon (x_{l})$. Then, the classifier C made predictions ${\tilde{y}}_{l}=C(f_{l})$ based on the feature map. Finally, the predictions ${\tilde{y}}_{l}$ were compared to the ground truth $y_{l}$ using cross entropy loss for supervision.

The training process of the unsupervised branch can be described as follows. Firstly, an unlabeled image $x_{u}$ was processed with two different transformations to get two images $x_{u1}$ and $x_{u2}$. These two images were then fed through the CNN (ε) to generate the feature maps $f_{u1}=\varepsilon (x_{u1})$ and $f_{u2}=\varepsilon (x_{u2})$. After that, the classifier C made predictions and based on the feature map, while the projector Φ change the feature dimension $\varphi_{u1}=\Phi (f_{u1})$ and $\varphi_{u2}=\Phi (f_{u2})$. Finally, we calculated the loss between the low dimension feature and the pseudo labels.

### 3.5. Evaluation metrics

In our experiments, we used Dice Similarity Coefficient (DSC) and Intersection-over-Union (IoU) to evaluate the performance of the proposed method for images segmentation.

The DSC and IoU can be described as follows:

(8) $$DSC=\frac{2\times |A\cap B|}{\left|A|+|B\right|}=\frac{2\times TP}{2\times TP+FP+FN}$$

(9) $$\;IoU=\frac{A\cap B}{A\cup B}=\frac{TP}{TP+FP+FN}$$

where A is the predicted set of pixels; B is the ground truth; TP represents the true positive; FP represents the false positive and FN represents the false negative. Note that IoU and DSC in the following tables and figures represent the average value of segmentation results from four classes, including background, LA, LV and myocardium.

### 3.6. Ablation study

**Figure 3:**
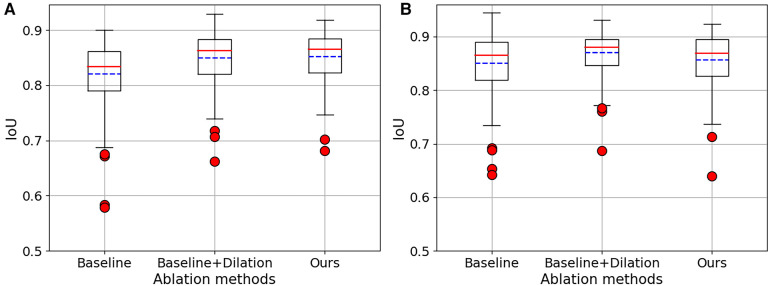
The variability of IoU about our ablation studies. (**A**) The results obtained from 2CH images. (**B**) The results obtained from 4CH images.

We conducted a series of ablation experiments to verify the contribution of each component in the proposed method. The ablation study was based on the full labeled data on CAMUS dataset. In our experiments, we first embed a standard u-net as the baseline. Then, we modified the u-net by adding dilated convolutions to the downsampling process and named it “Baseline+Dilation.” Finally, we added a projector to ”Baseline+Dilation” to complete proposed method, which we called “Baseline+Dilation+Projector.” The mean IoU and mean DSC results for each method were presented in Table 1 and Figure 2. Meanwhile, boxplots were employed to illustrate the variability of the mean Intersection over Union (IoU) for the aforementioned three methods in Figure 3.

**Table 1:** Statistical comparison of ablation studies on 2CH and 4CH images with full labeled training data.

Method	2CH				4CH			
	IoU	D(IoU)	DSC	D(DSC)	IoU	D(IoU)	DSC	D(DSC)
Baseline	0.811	0.004	0.893	0.002	0.854	0.003	0.919	0.002
Baseline + Dilation	0.847	0.003	0.916	0.001	**0.868**	0.002	**0.928**	0.001
Baseline + Dilation + Projector	**0.849**	0.002	**0.917**	0.001	**0.868**	0.002	**0.928**	0.001

The D(IoU) and D(DSC) represent the variance of the IoU and DSC scores, respectively.

The best results are achieved and highlighted by the bold values.

*Dilation:* In order to demonstrate the effectiveness of the dilated convolution applied in the u-net, we made a comparison between Baseline and Baseline with dilation (Baseline+Dilation). The experimental results shown that the model incorporated dilation (Baseline+Dilation) outperformed the one without dilation (Baseline), with an average IoU of 0.026 for 2CH images and 0.014 for 4CH images (Table 1).

*Projector:* In Table 1, we can see that incorporating a projector into the model improved the mean IoU from 0.847 to 0.849 in 2CH images. From Figure 2, we can find that the segmentation performance became better and more stable with the influence of projector in the last 30 epochs of the training.

### 3.7. Segmentation results and comparison with other methods

The proposed method was assessed for its ability to segment multiple cardiac structures, including LV, myocardium and LA. The segmentation performance was evaluated on the testing dataset of CAMUS dataset, comprising 40 patients with good or medium quality images and 10 patients with poor quality images.

While the CAC (Context Aware Consistency Network) method demonstrates strong performance in natural images segmentation, it falls short when it comes to accurately delineating cardiac structures in echocardiography (15). In certain cases, it may even incorrectly segment certain regions. Despite these limitations, CAC remains a widely employed semi-supervised technique for various image segmentation tasks, including those involving cardiac structures in echocardiography. Therefore, we compared the performance of our proposed method with CAC in order to demonstrate the superiority of our approach for cardiac segmentation. In addition, we compared our method with some supervised methods, including u-net and DeeplabV3+ with Resnet50 backbone, using all the labeled images available. To guarantee a fair comparison, we implemented all methods under the same conditions, including the same data augmentations and the same learning rate adjustment strategy.

The performance of each method has been presented in Tables 2 and 3, showcasing the results for the proposed method, CAC method, and other supervised methods in both the 2CH and 4CH views. From the tables, it was evident that the proposed method outperformed the other methods in terms of segmentation performance with 1/4, 1/2, and full labeled data. We also compared the number of parameters among u-net, DeeplabV3+, the proposed method, and the CAC method in Table 2, showing that our method had fewer parameters. Note that the values in the tables shown the maximum of the epochs we trained. The multi-structure segmentation performance of the proposed method has been presented in Table 4. We can see with the increase in the number of labeled images that take part in the training process, the Iou and DSC are improved.

**Table 2:** Segmentation performance comparison of IoU and DSC between the proposed method and other techniques.

Method	2CH				4CH				N	Params
	IoU	D(IoU)	DSC	D(DSC)	IoU	D(IoU)	DSC	D(DSC)		
DeeplabV3+	0.673	0.190	0.796	0.170	0.671	0.012	0.794	0.009	1/4	40.347MB
	0.70	0.014	0.816	0.012	0.713	0.011	0.825	0.009	1/2	
	0.786	0.005	0.876	0.003	0.818	0.003	0.896	0.002	Full	
U-net	0.780	0.007	0.872	0.005	0.796	0.006	0.882	0.003	1/4	17.267MB
	0.805	0.006	0.888	0.004	0.835	0.006	0.907	0.003	1/2	
	0.832	0.004	0.906	0.002	0.854	0.003	0.919	0.002	Full	
CAC	0.780	0.006	0.872	0.004	0.811	**0.002**	0.892	**0.001**	1/4	40.348MB
	0.784	0.005	0.875	0.003	0.824	**0.002**	0.900	**0.001**	1/2	
	0.787	0.004	0.877	0.002	0.836	0.003	0.908	**0.001**	Full	
Ours	**0.819**	**0.004**	**0.898**	**0.002**	**0.829**	0.003	**0.903**	0.002	1/4	17.268MB
	**0.838**	**0.003**	**0.911**	**0.002**	**0.857**	0.003	**0.921**	**0.001**	1/2	
	**0.849**	**0.002**	**0.917**	**0.001**	**0.868**	**0.002**	**0.928**	**0.001**	Full	

*N* represents the ratio of labeled images that we used. The *D*(IoU) and *D*(DSC) represent the variance of the IoU and DSC scores, respectively.

The best results are achieved and highlighted by the bold values.

**Table 3:** Segmentation performance comparison of precision and recall between the proposed method and other techniques.

Method	2CH				4CH				N
	P	D(P)	R	D(R)	P	D(P)	R	D(R)	
DeeplabV3+	0.809	0.010	**0.953**	**0.002**	0.847	0.005	0.950	**0.001**	1/4
	0.829	0.007	**0.956**	**0.001**	0.874	0.005	**0.971**	**0.001**	1/2
	0.896	0.002	**0.965**	**0.001**	0.908	0.002	0.957	**0.001**	Full
U-Net	0.847	0.004	0.870	0.004	0.881	0.004	0.926	0.002	1/4
	0.882	0.004	0.907	0.003	0.904	0.002	0.938	**0.001**	1/2
	0.902	0.002	0.924	0.002	0.911	0.002	0.939	**0.001**	Full
CAC	0.864	0.003	0.926	**0.002**	0.891	**0.002**	0.937	**0.001**	1/4
	0.880	0.002	0.927	0.002	0.903	**0.001**	0.940	**0.001**	1/2
	0.881	0.002	0.935	0.002	0.897	0.002	0.932	**0.001**	Full
Ours	**0.894**	**0.002**	0.927	**0.002**	**0.905**	**0.002**	**0.961**	**0.001**	1/4
	**0.908**	**0.001**	0.938	**0.001**	**0.921**	**0.001**	0.959	**0.001**	1/2
	**0.923**	**0.001**	0.948	**0.001**	**0.924**	**0.001**	**0.962**	**0.001**	Full

*N* represents the ratio of labeled images that we used. P represents precision and R represents recall. The D(P) and D(R) represent the variance of the precision and recall, respectively.

The best results are achieved and highlighted by the bold values.

**Table 4:** Segmentation performance of the proposed method on LV, LA and myocardium.

N		2CH				4CH			
		IoU	D(IoU)	DSC	D(DSC)	IoU	D(IoU)	DSC	D(DSC)
1/4	LV	0.824	0.007	0.904	0.003	0.832	0.008	0.908	0.003
	LA	0.806	0.014	0.893	0.007	0.837	0.012	0.911	0.011
	Myocardium	0.694	0.012	0.819	0.007	0.697	0.013	0.809	0.008
1/2	LV	0.836	0.007	0.911	0.003	0.865	0.005	0.928	0.002
	LA	0.830	0.011	0.907	0.005	0.843	0.014	0.915	0.012
	Myocardium	0.726	0.009	0.841	0.005	0.748	0.008	0.856	0.004
Full	LV	0.848	0.003	0.917	0.001	0.877	0.004	0.934	0.002
	LA	0.842	0.010	0.915	0.005	0.854	0.015	0.921	0.012
	Myocardium	0.744	0.005	0.851	0.002	0.766	0.006	0.867	0.003

*N* represents the ratio of labeled images that we used.

**Figure 4:**
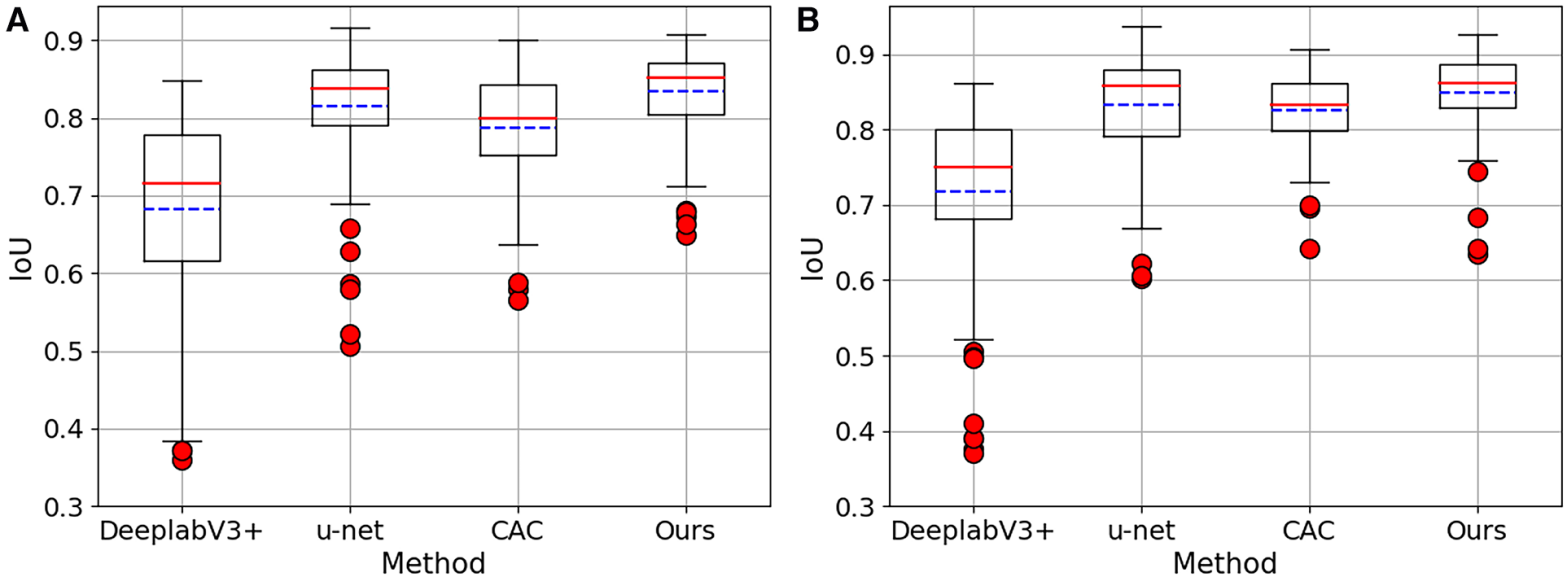
The Boxplots show the IoU values for both 2CH and 4CH images, using the four methods mentioned in Table 2. These boxplots specifically showcase the outcomes achieved when employing 1/2 labeled images. (**A**) The results obtained from 2CH images. (**B**) The results obtained from 4CH images.

**Figure 5:**
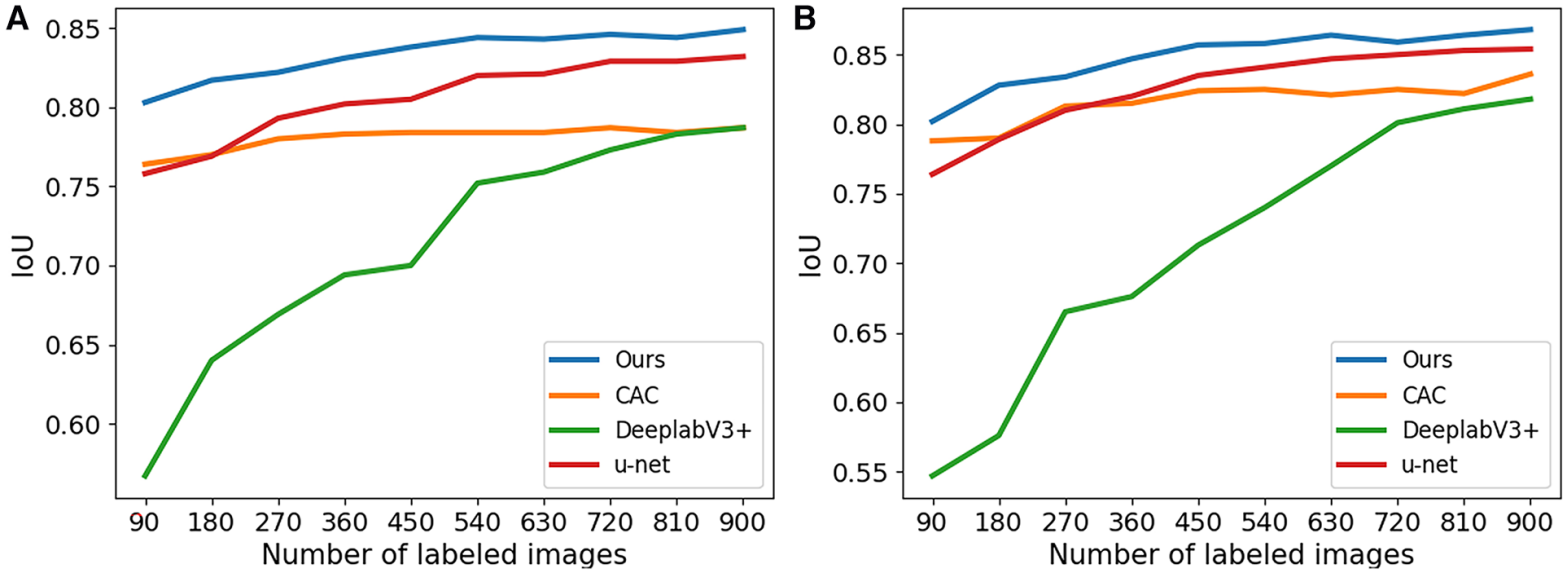
The IoU for segmentation results of the proposed method trained with different numbers of labeled images. (**A**) The results obtained from 2CH images. (**B**) The results obtained from 4CH images.

In Figure 4, boxplots have been used to visually represent the range of variation in IoU values achieved by the four methods mentioned earlier, where 1/2 labeled images were employed for training. We can see our proposed method achieved lower variation and higher mean IoU for for both 2CH and 4CH images, in comparison to the other methods. In addition, Figure 5 illustrated the trends of mean IoU as the number of labeled images increases. It was observed that as the number of labeled images utilized in the training process increased, the mean IoU also improved. Notably, the proposed method consistently outperformed the other three methods in terms of segmentation accuracy across all increments of labeled data.

**Figure 6:**
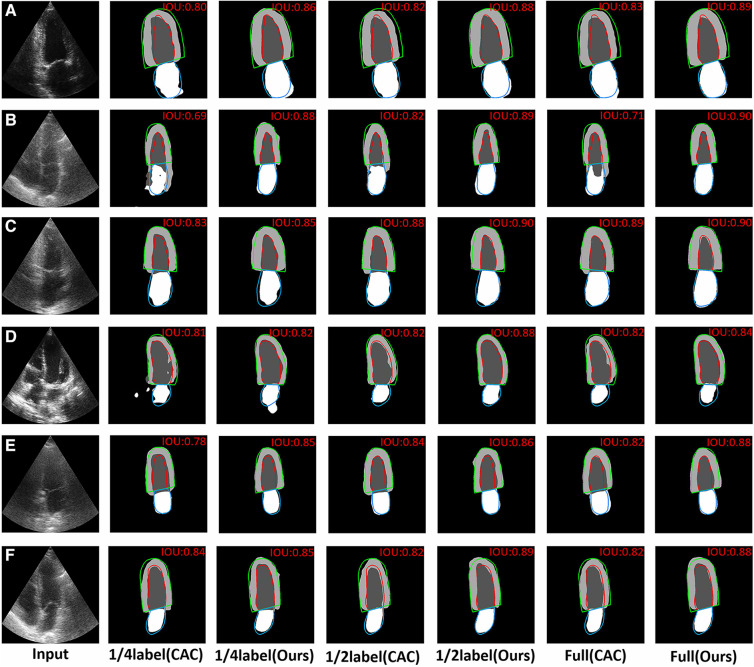
Visual comparison of CAC method and the proposed method with 1/4labeled, 1/2labeled and full labeled data on 2CH and 4CH images. The red, green and blue lines represent the ground truth. The value of IoU is marked on the right upper corner of each image. (**A**,**B**) The visual comparison of the performance about discriminating the borders of myocardium on 2CH images and 4CH images. (**C**,**D**) The visual comparison of the probability of segmentation errors in some regions on 2CH images and 4CH images. (**E**,**F**) The visual comparison of the performance about the identification of the ventricle and myocardium location.

The typical visual segmentation result of CAC method and the proposed method were shown in Figure 6, where the colorful line represents the ground truth. In Figures 6A,B, it shown that the proposed method could discriminate the borders of myocardium better than CAC method. Figures 6C,D shown that the proposed method could reduce the probability of segmentation errors in each region of the images. Figures 6E,F shown that the proposed method achieved a better identification of the ventricle and myocardium location than CAC method.

**Figure 7:**
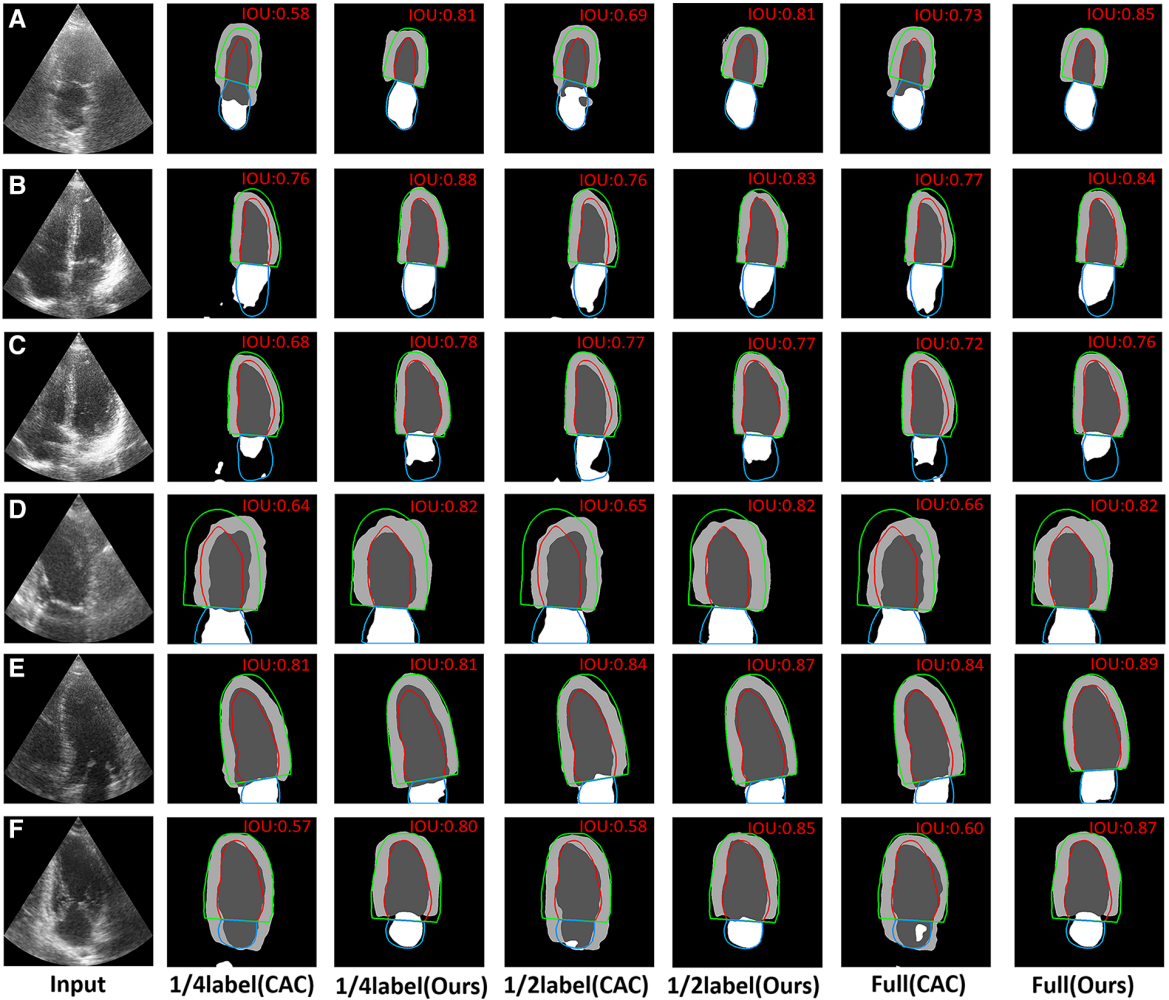
Visual comparison of CAC method and the proposed method on 6 typical challenging images. The red, green and blue lines represent the ground truth. The value of IoU is marked on the right upper corner of each image. (**A**–**C**) The visual comparison of the performance on some typical low contrast images. (**D**) The visual comparison of the performance on images where the complete cardiac structures are not present. (**E**,**F**) The visual comparison of the performance on images that have no a clear border between the ventricle and the atrium.

In Figure 7, we shown certain challenging cases with poor image quality. The IoU values are presented in the upper right corner of each segmentation result. It becomes evident that the proposed method surpassed limitations of echocardiography more effectively. Specifically, Figures 7A–C demonstrate that the proposed method outperformed the CAC method in mitigating the disadvantage of low contrast in echocardiography. Additionally, Figure 7D showcases that the proposed method achieved superior heart location identification compared to the CAC method in images where complete cardiac structures are not present. Moreover, Figures 7E,F indicate that the proposed method reduced the likelihood of segmentation errors in regions lacking clear boundaries between the ventricle and the atrium.

## 4. Conclusion

In this paper, we proposed a semi-supervised method to segment the cardiac structures with echocardiography. The proposed method first applied contrastive learning strategy into cardiac structure segmentation, allowing for effective use of unlabeled data. The network was able to mitigate the adverse effects of low contrast, incomplete cardiac structures and unclear boundaries in certain aspects of echocardiography. A lot of experiments conducted on the CAMUS dataset shown that the proposed network can effectively employ unlabeled data for the automatic segmentation of multiple structures, resulting in outstanding performance. This advancement contributes significantly to the diagnosis and screening of cardiovascular diseases (CVD), and also reduce the burden of doctors in assessing echocardiography.

## Data Availability

Publicly available datasets were analyzed in this study. This data can be found here: https://www.creatis.insa-lyon.fr/Challenge/camus/databases.html.
